# Non-Coding RNAs and Their Role in Maintaining Epidermal Homeostasis

**DOI:** 10.3390/cimb47110924

**Published:** 2025-11-06

**Authors:** Daniil D. Romashin, Tatiana V. Tolstova, Alexander L. Rusanov, Natalia G. Luzgina

**Affiliations:** Institute of Biomedical Chemistry, Moscow 119121, Russia

**Keywords:** skin, epidermis, microRNA, lncRNA, circRNA, psoriasis, wound healing, p63, chronic wounds

## Abstract

In recent decades, there has been a significant amount of research on the biological role of non-coding RNAs (ncRNAs) in both normal and pathological conditions. Specifically, a growing body of evidence suggests that ncRNAs play a crucial role in maintaining epidermal homeostasis. These ncRNAs are involved in regulating epidermal differentiation and wound healing, as well as in pathological skin conditions, such as psoriasis and chronic wounds. The discovery of mechanisms such as RNA interference and other modes of action of ncRNAs has led to the development of novel therapeutic strategies, where ncRNAs could serve as targets, therapeutic agents, or diagnostic markers. This review explores the role of different classes of ncRNAs in the epidermis under normal and abnormal conditions, the mechanisms by which ncRNAs interact with other modulators of epidermal homeostasis, and the current state of ncRNA-based therapy.

## 1. Introduction

The human skin acts as a protective barrier that interacts with both biotic and abiotic environmental factors. It protects the body from harmful external elements and helps prevent fluid and nutrient loss [1]. The skin consists of two main parts: the interfollicular epidermis (IFE) and the hair follicles. Most of the IFE cells (about 90%) are keratinocytes, which form multiple layers of cells, including the stratum basale, stratum spinosum, stratum granulosum, and stratum corneum [2]. Keratinocytes are essential for forming the epidermal barrier. This process begins in the granular layer of the epidermis, where cells accumulate proteins in their cytoplasm, forming horny envelopes. These proteins create dense intercellular connections, reinforcing the epidermal barrier [2].

The formation of the stratum corneum results from the terminal differentiation of keratinocytes. During this process, basal-layer cells lose contact with the basement membrane and migrate to the upper epidermal layers. These cells are characterized by the absence of nuclei and are filled with keratin filaments [3]. Epidermal differentiation is associated with the loss of the proliferative potential in basal cells and the continuous renewal of the outermost layer, or stratum corneum, as keratinocytes undergo terminal differentiation. Differentiation is accompanied by coordinated transcriptional and epigenetic changes.

Disruption of this balance occurs when the epidermis is damaged by external factors (infections, mechanical or thermal injury) or when inflammation of different origins develops in the dermis (often allergic or autoimmune inflammation). This causes activation of keratinocyte migration, growth, and proliferation to repair the damage, leading to changes in the epidermis’s normal structure and functional defects in the stratum corneum. Keratinocytes themselves play an active role in the inflammatory response by secreting various cytokines and chemokines (e.g., IL-33, IFNγ, IL-17A), recognizing pathogens, releasing proteases and inhibitors, and performing other functions aimed at restoring normal epidermal function [4].

The complex process of maintaining epidermal homeostasis is regulated by transcription factors, epigenetic regulators, signaling molecules, and receptors. These include proteins such as p53, NEDD4–1, the AP-2a and AP-2b families, PDK1, among others [5,6,7,8,9]. Recent studies have shown that non-coding RNAs (ncRNAs) also play an important role in maintaining epidermal homeostasis and are involved in regulating epidermal differentiation and wound healing, as well as in pathological skin processes such as psoriasis and chronic wounds [10,11,12]. This review aims to systematize current knowledge on the role of different classes of ncRNAs in regulating epidermal differentiation under physiological and pathological conditions, including chronic wounds, which are characterized by impaired re-epithelialization and persistent inflammation.

## 2. Methodology

A focused literature search was conducted using the PubMed, Scopus, and Web of Science databases. The search terms included “non-coding RNA”, “microRNA”, “miRNA”, “long non-coding RNA”, “lncRNA”, “circular RNA”, “circRNA”, “epidermal differentiation”, “p63”, “wound healing”, “diabetic wounds”, “chronic wounds”, “psoriasis”, “ASO”, “siRNA therapeutics”, and “ncRNA therapeutics”. Only peer-reviewed English-language studies on ncRNAs in epidermal biology, regeneration, or pathology were included in the search. Conference abstracts and irrelevant models were excluded from the search. After screening, approximately 150 references were selected, with about 35% published between 2023 and 2025 to ensure the inclusion of recent findings in the field.

## 3. ncRNAs: Classification and Functions

It has long been known that a small percentage of the human genome consists of protein-coding genes, while the majority of genomic sequences are transcribed into ncRNAs that do not encode proteins but regulate gene expression at multiple levels. These ncRNAs help fine-tune the expression of genes, ensuring that they are expressed at the right time and in the right amount [10,13].

Functionally, ncRNAs are divided into structural and regulatory types. Structural ncRNAs include small nuclear RNAs (snRNAs) and small nucleolar RNAs (snoRNAs), which modify ribosomal and transfer RNAs, respectively [11,12]. Regulatory ncRNAs are classified by length into short (<200 nucleotides) and long (>200 nucleotides) classes. The first class includes PIWI-interacting RNAs (piRNAs) [14], small interfering RNAs (siRNAs), and microRNAs (miRNAs). The other class includes long non-coding RNA (lncRNAs), circular RNAs (circRNAs), and recently discovered enhancer RNAs (eRNAs) [10].

In the epidermis, three regulatory classes—miRNAs, lncRNAs, and circRNAs—dominate functional control of keratinocyte biology. They regulate keratinocyte proliferation, differentiation, migration, apoptosis, and the response to environmental stress [15,16,17].

MicroRNAs are small (20–25 nucleotides) double-stranded RNA molecules that are formed through the processing of primary miRNA (pri-miRNA) transcripts by enzymes DROSHA and DICER [17]. In the cytoplasm, miRNAs are bound by Argonaute (AGO) family proteins. These proteins induce the miRNAs to bind to a complementary sequence on the 3′UTR (untranslated region) of the target mRNA. This binding leads to the formation of an RNA-induced silencing complex (RISC) [15]. This interaction results in either mRNA degradation or translational repression, leading to inhibition of the target gene [18]. The number of targets for each miRNA can vary, ranging from tens to hundreds of genes, due to the relatively low degree of specificity in its interaction with the target mRNA sequence. In addition, miRNAs duplexes consist of two strands: a guide strand and a passenger strand (often denoted by “*” in the literature). Although the strand with lower 5′-end thermal stability is typically incorporated into RISC, both strands may function as regulatory guides [15,19].

LncRNAs are a group of non-coding RNAs that are longer than 200 nucleotides and are heterogeneous in terms of their origin, location in the genome, and functions [16,20]. They can suppress gene expression through several mechanisms, including: (I) disruption of the transcription process and interference with transcription factors (TFs) and RNA polymerase II (Pol II); (II) participating in chromatin remodeling, reducing the availability of the chromatin; (III) entering into complexes with various proteins and other RNA molecules [21].

CircRNAs have a unique, covalently closed-loop structure with a median length of approximately 700–800 nucleotides [22], with a significant proportion (>20%) exceeding 500 nucleotides [23]. CircRNAs are generated from precursor mRNA as a result of the activity of RNA polymerase II. The 3′ and 5′ ends of the transcript are spliced together to form a circular structure (back-splicing). CircRNAs do not contain 5′-caps or 3′-poly(A) [24]. CircRNAs are able to regulate the expression of genes by binding to RNA polymerase II. Functionally, circRNAs can act as “sponges” for miRNAs, preventing them from binding their target mRNAs, or as protein-binding molecules that regulate transcription through the competing endogenous RNA (ceRNA) mechanism [25].

Most physiological processes, such as embryogenesis, morphogenesis, and cell differentiation, are regulated by ncRNAs to a certain degree. Aberrant regulation of ncRNAs, in turn, may be involved in the development of various diseases, including different types of cancer, neurodegenerative and immune disorders, cardiovascular diseases, and other pathologies [26,27].

Other ncRNA types (e.g., piRNAs, snoRNAs) are also expressed in the skin, but their roles in epidermal homeostasis remain less mechanistically defined. Therefore, this review focuses primarily on miRNAs, lncRNAs, and circRNAs, reflecting the strongest current evidence linking these ncRNA classes to epidermal function and dysregulation in skin disorders.

## 4. ncRNAs Regulating Epidermal Differentiation

The process of epidermal differentiation involves precise regulation of gene expression, and miRNAs play a significant role in this process. For example, miR-203 is known as the master regulator of epidermal homeostasis. Its expression is suppressed in proliferating keratinocytes, but increases markedly as cells migrate towards the suprabasal layers of the epidermis [28]. MiR-203 promotes keratinization by targeting the mRNAs of genes associated with keratinocyte stem cell maintenance, such as *TP63* (Tumor protein p63), *SKP2* (S-phase kinase-associated protein 2), *MSI2* (Musashi RNA-binding protein 2), *SRC* (Proto-oncogene tyrosine-protein kinase Src) and *RAPGEF1* (Rap guanine nucleotide exchange factor 1) [29,30,31,32]. In addition, survivin (BIRC5, Baculoviral IAP repeat-containing protein 5), an inhibitor of apoptosis in basal keratinocytes, has recently been shown to be a direct target of miR-203, further supporting epidermal homeostasis [33,34].

During the cultivation of primary human keratinocytes in 2D and 3D models, miR-141-3p was also found to be highly expressed in differentiating keratinocytes, along with miR-203 [29]. In the same study, it was found that overexpression of miR-141 results in a decrease in the proliferation of keratinocytes and the induction of key markers of epidermal differentiation: *IVL* (Involucrin), *LOR* (Loricrin), *KRT1/10* (Keratin 1/10), *FLG* (Filaggrin) and *TGM1* (Transglutaminase 1). Also, miR-203, along with miR-198, targets *CCND2* (Cyclin D2), which is essential for cell cycle progression [29,35]. Another example of a miRNA involved in the regulation of epidermal differentiation is miR-184. This miRNA induces epidermal differentiation by directly repressing the expression of keratin 15 (K15) and activating the Notch signaling pathway [36].

A role in maintaining the stem potential of keratinocytes has also been demonstrated for miR-148a, which modulates ROCK1 (Rho-associated protein kinase 1) and ELF5 (E74-like factor 5) expression. Inhibiting miR-148a leads to upregulation of *KRT1*, *KRT10* and *IVL* [37]. MiR-130b-3p inhibits keratinocyte differentiation by suppressing Dsg1 (Desmoglein-1) expression. However, its effect is counteracted by lncRNA H19 (H19 Imprinted Maternally Expressed Transcript), which functions as a “sponge” that binds miR-130b-3p and inhibits its activity [38].

Two lncRNAs, DANCR (Differentiation Antagonizing Non-Coding RNA) and TINCR (Terminal Differentiation-Induced Non-Coding RNA), regulate keratinocyte differentiation through distinct mechanisms. DANCR decreases as epidermal differentiation progresses, supporting the phenotype of basal keratinocytes by suppressing the transcription factors MAF and MAFB (MAF bZIP Transcription Factor Family) [39,40]. TINCR, on the other hand, is a regulator of keratinization through the stabilization of mRNA encoding differentiation genes, including MAF, MAFB, and STAU1 (Staufen RNA-binding protein 1) [39,40].

HOXC13-AS sequesters COPA (Coatomer subunit alpha), a subunit of the coatomer complex, disrupting retrograde Golgi-to-ER transport and inducing ER stress (Endoplasmic reticulum stress), which promotes keratinocyte differentiation [41].

Lee et al. have discovered the epigenetic mechanism through which lncRNA Pvt1 supports the stem potential of epidermal progenitor cells [42]. In particular, it has been shown that the N6-methyladenosine modification (m6A) of Pvt1 stabilizes its binding to c-Myc (Myc proto-oncogene protein), which helps to maintain proliferation. On the other hand, demethylation of Pvt1 enhances epidermal differentiation [42].

Another example of lncRNAs that regulate keratinocyte differentiation at the epigenetic level is LINC00941 [43]. Initially, LINC00941 was identified as a positive regulator of basal keratinocyte proliferation and a regulator of premature keratinocyte differentiation. This is due to its ability to suppress the expression of SPRR5, a newly discovered positive regulator of epidermal differentiation [41]. Later, it has been shown that LINC00941 directly interacts with multiple components of nucleosome remodeling and deacetylase (NuRD) complex, modulating its binding to chromatin and repressing the *EGR3* (Early Growth Response 3), which acts as a positive regulator of keratinocyte differentiation. During keratinocyte differentiation, LINC00941 abundance decreases, releasing the *EGR3* expression and promoting keratinocyte differentiation [43].

Differentiated keratinocytes exhibit a significant increase in the expression of circRNAs expression, including circCTEN (circular CTEN/TNS4 RNA; Tensin 4), circDLG1 (circular DLG1 RNA; Discs Large MAGUK Scaffold Protein 1), circHECTD1 (circular HECTD1 RNA; HECT Domain E3 Ubiquitin Ligase 1) and circHOMER1 (circular HOMER1 RNA; Homer Scaffold Protein 1), compared with epidermal stem cells [44]. This expression pattern suggests that circRNAs contribute to the execution and stabilization of the late epidermal differentiation program. It has been shown that circRNAs transcribed from the ZNF91(Zinc finger protein 91) gene have 24 binding sites for miR-23b-3p, which plays a role in keratinocyte differentiation [44,45]. The presence of multiple miRNA-binding sites indicates that circZNF91 may act as a miRNA sponge, modulating post-transcriptional control of epidermal gene expression.

Overall, circRNAs appear to fine-tune keratinocyte differentiation by regulating miRNA availability and supporting transcriptional stability in the upper epidermal layers.

### p63-ncRNA Regulatory Circuitry

**Figure 1:**
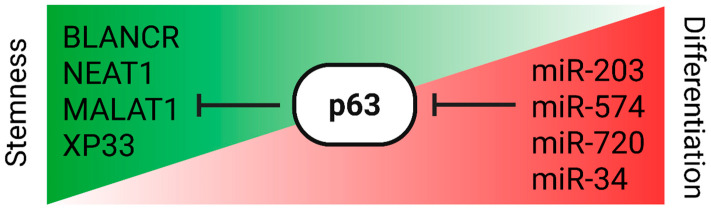
The crosstalk between p63 and non-coding RNAs (ncRNAs) in epidermal differentiation. The expression of microRNAs (miRNAs) miR-203, miR-574, miR-34 and miR-720 increases during the differentiation of keratinocytes. These microRNAs target the transcription factor p63 (encoded by *TP63*), thus unblocking the keratinization program. P63 is involved in maintaining the stemness of keratinocytes, including through the regulation of non-coding RNA. P63 suppresses the transcription of long non-coding RNAs (lncRNAs) NEAT1 (nuclear paraspeckle assembly transcript 1) and MALAT1 (metastasis-associated lung adenocarcinoma transcript 1) at the epigenetic level, while activation of these lncRNAs promotes differentiation. LncRNAs BLANCR (basal layer-associated non-coding RNA) and XP33 (*LINC00302*) are direct targets of p63 and their activation correlates with a decrease in p63 levels during epidermal differentiation.

Interactions between ncRNA and *TP63*, which encodes the protein p63, the master regulator of epidermal differentiation [46], are of particular interest. The isoforms of p63 have a complex and multidirectional effect on epidermal differentiation, interacting harmoniously to finely regulate it. The TAp63 isoform supports the stratification of keratinocytes, while ΔNp63 plays a key role in supporting the “stemness” of basal keratinocytes and restricting the keratinization program [47]. Being a transcription factor, p63 is involved not only in the regulation of protein-coding genes, but is also a direct transcriptional regulator of some ncRNAs (Figure 1). A transcriptomic study on primary human keratinocytes with p63 knockdown showed that lncRNA XP33 (LINC00302) is a direct target of ΔNp63a and accumulates during late stages of differentiation. Conversely, knocking down XP33 led to a repression of the keratinization program and a decrease in the level of LCE2D (Late cornified envelope protein 2D), a marker for the late stage of epidermal differentiation [48]. Other p63-regulated lncRNAs include NEAT1 (Nuclear paraspeckle assembly transcript 1) and MALAT1 (Metastasis-associated lung adenocarcinoma transcript 1), whose expression suppresses epidermal differentiation [49]. In the absence of differentiation stimuli, ΔNp63 binds to HDAC1/2 (Histone deacetylase 1/2) and recruits them to the NEAT1 and MALAT1 promoters. This results in histone deacetylation and suppression of their transcription. However, under the influence of differentiation signals, this mechanism becomes weaker, leading to the activation of NEAT1/MALAT1 expression. The activated NEAT1/MALAT1 then positively regulates the expression of genes involved in differentiation [50,51].

Another example is lncRNA BLNCR (Basal cell-specific lncRNA), whose expression decreases significantly upon induction of keratinocyte differentiation. The expression of BLNCR is directly regulated by p63 and AP-1 (Activator protein-1) [52].

Beyond miR-203, ΔNp63 is also targeted by miR-574 and miR-720, whose expression increases during keratinocyte differentiation [51,53].

In addition, the relationship between p63 and microRNAs may be complementary and involve other factors. In particular, p53 is of great interest in this context. In human keratinocytes, ΔNp63 shows dominant-negative activity [54]. In the epidermis, ΔNp63 and p53 have opposite functions, but both compete for some targets. For example, they both bind to *CDKN1A* (p21) and SFN (14-3-3σ) [55]. Interestingly, suppression of p53 results in a decrease in the level of miR-203 in human keratinocytes [32]. In addition, p53 acts as a transcriptional activator for the miR-34 family, leading to significant changes in the transcriptome, suppression of the cell cycle, and induction of apoptosis [56]. P63, in contrast, inhibits miRNAs of this family (miR-34a/c) by binding to elements of the p53 response, contributing to the progression of the cell cycle [57].

Since wound repair requires reactivation of keratinocyte proliferation and migration that are normally suppressed during terminal differentiation, ncRNAs regulating keratinocyte fate also play essential roles in epidermal regeneration. Therefore, the following section focuses on ncRNA-mediated mechanisms in wound healing.

## 5. NcRNAs in Regenerative Repair

### 5.1. Normal Wound Healing

The skin, as a barrier tissue, is frequently exposed to aggressive environmental factors and serves as a platform for inflammatory processes, which can lead to tissue damage and disruption of integrity. Reparative regeneration aims to repair these defects and restore the cellular composition and functionality of the tissue through balanced programs. Depending on wound severity, regeneration may occur with full restoration or result in fibrosis and scarring.

**Figure 2:**
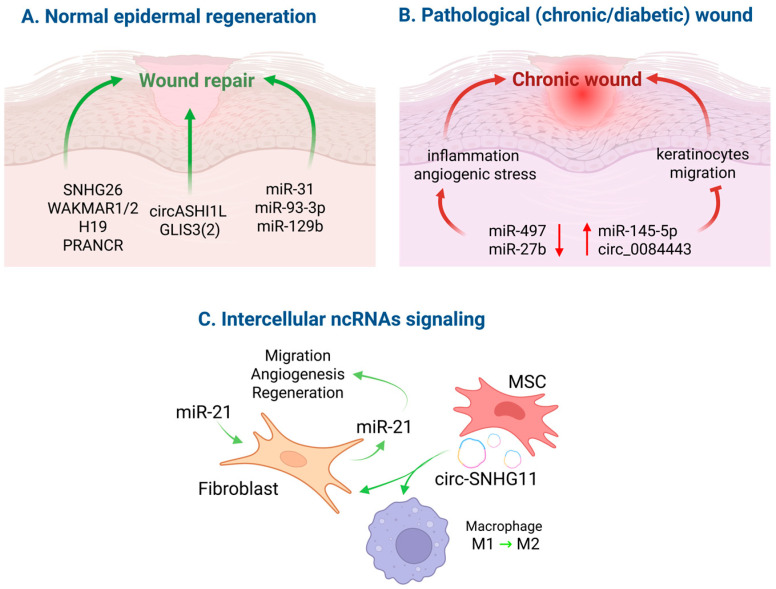
The role of non-coding RNAs (ncRNAs) in regenerative repair and chronic wounds: (**A**) Normal epidermal regeneration: Pro-reparative ncRNAs, such as miR-31 and miR-93-3p, and their associated transcription factors, including ΔNp63 and STAT3, promote the migration of keratinocytes and re-epithelialization; (**B**) Chronic or diabetic wounds: Impaired closure due to persistent inflammation and reduced angiogenesis associated with upregulation of miR-145-5p and circ_0084443, downregulation of miR-497 and miR-27b involved in the NRF2 pathway; (**C**) Intercellular RNA signaling: Exosomal miR-21 promotes fibroblast activation and angiogenesis while circ-SNHG11 from MSCs promotes macrophage polarization towards M2 phenotype.

Keratinocytes are central to epidermal repair, particularly when the barrier is breached. Despite decades of research, the precise molecular mechanisms coordinating their behavior remain incompletely defined [58]. Keratinocyte proliferation replenishes damaged areas with new cells, while terminal differentiation re-establishes the stratum corneum and barrier [58,59]. At the wound edge, transcriptomic reprogramming results in dynamic shifts in gene expression, enabling migration proximally and differentiation distally [60,61]. It is known that ΔNp63 and STAT3 (Signal transducer and activator of transcription 3) play a major role in coordinating the regeneration process. Other important components of this network include p53, AP-1, Notch, cMYC, and EGFR [58]. Consequently, ncRNAs that control keratinocyte fate in homeostasis also regulate regenerative responses (Figure 2).

Recent studies highlight the importance of regulatory ncRNAs in wound healing. They modulate the processes of inflammation, proliferation, and remodeling at the site of damaged skin. For example, miR-34a and miR-34c have been shown to enhance the production of pro-inflammatory cytokines by keratinocytes. The introduction of miR-34a mimics into the skin wounds of mice has been shown to slow down their healing [62].

In addition, miRNAs have “pro-repair” properties (Figure 2A). Specifically, miR-31 has been shown to concentrate at the edge of a wound, and when it is knocked out in the epidermis, it leads to slower healing by reducing keratinocyte proliferation and migration. Thus, miR-31 plays a role in re-epithelialization [63]. Similarly, in a mouse model, miR-93-3p was shown to increase keratinocyte proliferation and migration through the ZFP36L1 (Zinc finger protein 36-like 1)/ZFX (Zinc finger protein X-linked) pathway, emphasizing that the miRNA network fine-tunes “epithelial plasticity” [2].

Extracellular vesicles (EVs) and their contents also play an important role in intercellular RNA signaling. Previous studies have shown that keratinocyte vesicles enriched with miR-21 directly stimulate fibroblast migration and angiogenesis, as well as accelerate wound closure in diabetic rats. This effect is associated with the activation of the MAPK/ERK signaling pathway in fibroblasts, contributing to their function and enhanced angiogenesis when exposed to endothelial cells [64]. Additionally, it has been recently confirmed that dermal fibroblasts secrete miR-21, which triggers a signaling cascade leading to the release of IL-1β (Interleukin-1β) by dermal fibroblasts. This, in turn, stimulates the movement and growth of skin cells, speeding up the wound healing process [65].

The role of lncRNAs in epidermal wound healing has been studied less extensively than miRNAs, but recent research has identified more and more lncRNAs involved in repair processes. Today, lncRNAs such as H19, PRANCR (Proliferation and differentiation-regulated long non-coding RNA), GAS5 (Growth arrest-specific transcript 5), MALAT1 (Metastasis-associated lung adenocarcinoma transcript 1), and HOTAIR (HOX transcript antisense RNA) are well-known examples, as reviewed in references [63,64].

Notably, MALAT1 has been shown to play a role in regulating the epithelial–mesenchymal transition (EMT) in keratinocytes [66,67]. It has been demonstrated that MALAT1 positively regulates the TGF-β1(Transforming Growth Factor β1)-induced EMT in HaCaT cells by binding and inactivating miR-205, which inhibits EMT by targeting the ZEB1 (Zinc finger E-box-binding homeobox 1) gene [66]. MALAT1, therefore, facilitates keratinocyte activation and migration at the wound edge (Figure 2A).

Newly identified lncRNAs, such as WAKMAR1 (Wound And Keratinocyte Migration-Associated lncRNA 1) and WAKMAR2, also play roles in wound healing. It has been shown that WAKMAR1 regulates the migration of keratinocytes by interacting with E2F1 (E2F transcription factor 1), while WAKMAR2 inhibits the production of inflammatory chemokines, such as CXCL8/IL8 (Interleukin-8), CCL20, and CXCL5 [68,69].

A recent discovery is that lncRNA SNHG26 (Small Nucleolar RNA Host Gene 26) plays a role in regulating the transition of keratinocyte precursors from an inflammatory state to proliferation, through its interaction with the ILF2 (Interleukin enhancer-binding factor 2) transcription factor (Figure 2A). When SNHG26 is silenced, genes associated with inflammation, such as IL6, IL8, and CCL20, are suppressed. Conversely, overexpression of SNHG26 leads to the opposite effect, promoting proliferation [70].

Another example is lncRNA H19, which is involved in the proliferation and migration of keratinocytes. Its decrease suppresses the degradation of the extracellular matrix through the miR-17-5p/RUNX1 (Runt-related transcription factor 1) pathway [71].

Together, these lncRNAs assist in orchestrating inflammatory resolution and initiation of re-epithelialization during normal repair.

Transcriptomic analysis of healthy and wounded keratinocytes has shown that circRNAs also contribute to the process of reparative epidermal regeneration [72]. In particular, circRNAs hsa-TNFRSF21_0001 and hsa-CHST15_0003 are highly expressed in keratinocytes from trophic ulcers. The knockdown of hsa-TNFRSF21_0001 leads to activation of genes involved in differentiation, EMT, and cell adhesion. Similarly, the knockdown of hsa-CHST15_0003 leads to activation of genes related to migration. Furthermore, the depletion of both circRNAs results in the repression of genes linked to the G2/M checkpoint [72]. Wang and colleagues found that circASHI1L (circular Ash1L RNA) is induced by TGF-β in wound keratinocytes (Figure 2A). It interacts with miR-129b and leads to its stabilization. This, in turn, leads to increased cell migration, proliferation, and acceleration of epithelialization [73].

Another example is circGLIS3(2) (circular GLIS Family Zinc Finger 3 RNA), which is expressed in dermal fibroblasts and leads to the activation of the secretion of extracellular matrix (ECM) components and wound closure [74]. Collectively, circRNAs complement miRNAs and lncRNAs by coordinating keratinocyte activation, ECM remodeling and cell-migration responses during normal repair.

### 5.2. Chronic and Diabetic Wounds

In the case of chronic, particularly long-term, non-healing wounds—particularly diabetic ulcers, the normal process of reparative regeneration is disrupted. This disruption is associated with increased activity of ncRNA, which leads to persistent inflammation, oxidative stress, and microcirculatory dysfunction. Such changes prevent the normal transition from inflammation to proliferation and re-epithelialization. Previous studies have shown that miR-497 expression is decreased in diabetic wounds in mice, and that local injections of a miR-497 mimic accelerate wound closure and reduce levels of pro-inflammatory cytokines such as TNF-α (Tumor necrosis factor-α), IL-6, and IL-1β (Figure 2B) [75]. This study confirms the anti-inflammatory and pro-repair potential of targeted miRNA therapy. In contrast, miR-145-5p has been found to be elevated in the sites of diabetic wounds, inhibiting their healing in models of diabetic foot (DF) disease. This effect occurs through the inhibition of PDGF-D (platelet-derived growth factor D). Suppressing miR-145-5p can restore the normal kinetics of regenerative repair, justifying the use of anti-miR-145-5p therapy for diabetic wounds [76].

Angiogenic stress is a key factor in the chronicity of wounds in diabetes. Therefore, in diabetic foot ulcers (DFUs), the activity of NRF2 (Nuclear factor erythroid 2-related factor 2) is suppressed, and the regulatory miR-27b plays a role in disrupting the NRF2-mediated angiogenesis pathway. It has been demonstrated that the pharmacological modulation of NRF2 in patients is correlated with an improvement in ulcer condition [77]. On the other hand, Nrf2 contributes to epidermal integrity through microRNA-regulated pathways. Specifically, Nrf2 directly targets clusters of miR-29A and miR-29B1, leading to an increase in the expression of these miRNAs. Both miR-29a and miR-29b-1 target the mRNA of *DSC2* (Desmocollin-2), which results in its sequestration and a decrease in its expression. The decrease in *DSC2* expression then leads to impaired hyper-adhesive desmosome formation and disruption of epidermal homeostasis [78]. Interestingly, the miR-29 family (miR-29a/b/c) contributes to epidermal remodeling by targeting a broad range of cell-adhesion-related genes in keratinocytes and dermal fibroblasts [79].

At the level of clinical biomarkers in patients with diabetes mellitus, an increase in miR-222-3p was also detected in peripheral blood and ulcer margins, which is associated with the risk and prognosis of DFU. This finding suggests that circulating miRNAs may be useful as screening and prognostic markers for DFU [80]. In addition, the clinical significance of miR-103 at the ulcer margin has also been indicated. In particular, its level has been negatively correlated with the rate of epithelialization [81].

In chronic wounds, circRNAs play a role in preventing epithelialization. Therefore, the authors have previously shown that hsa_circ_0084443 levels are elevated in human chronic DFU tissues [82]. This circRNA inhibits the migration and/or proliferation of keratinocytes, and its level decreases during normal wound healing. This makes circ_0084443 a promising target for the treatment of chronic ulcers. CircRNAs also play a role in immune responses. In particular, adipose-derived mesenchymal stem cells (AD-MSCs), under hypoxic conditions, can secrete exosomes carrying the circular RNA circ-SNHG11 (circular Small Nucleolar RNA Host Gene 11), which convert macrophages to M2 phenotype and accelerate the closure of diabetic wounds in mice (Figure 2C) [83]. These approaches have the potential to be highly effective in the targeted treatment of chronic inflammation in patients with diabetes.

### 5.3. ncRNA-Based Therapeutic Strategies

Collectively, these findings provide a comprehensive understanding of the role of ncRNAs in skin wound healing. Normal reparative regeneration relies on precise coordination between different cells and signaling molecules. Pro-reparative microRNAs (miR-31 and miR-93-3p), as well as certain lncRNAs, such as SNHG26, coordinate the transition from inflammation to proliferation and epithelialization. On the other hand, excess activity of inhibitory regulators, such as miR-34 and some circRNAs, disrupts this phase change. In diabetic conditions, chronic inflammation, oxidative stress, and vascular dysfunction disrupt the normal regulation of ncRNA networks. The expression of repair-inhibiting microRNAs, such as miR-145-5p, increases, while the level of pro-reparative miRNAs, like miR-497, decreases. Additionally, “inhibitory” circRNAs, such as circ_0084443, are activated. The clinical profiles of circulating microRNAs acquire prognostic significance. This, in turn, influences therapeutic approaches: the use of local mimics and siRNA inhibitors, the targeting of pathological circRNAs, the delivery of therapeutic ncRNAs using exosomes (including the hypoxic “priming” of donor cells), and the use of hydrogel systems (Figure 2C).

## 6. The Landscape of Non-Coding RNAs in Psoriatic Keratinocytes

Psoriasis is a chronic skin condition characterized by recurrent inflammation in the skin’s dermis, excessive proliferation of keratinocytes, and impaired epidermal differentiation. This leads to a disruption of the skin’s normal homeostasis [84]. The IL-23/IL-17A signaling pathway plays a crucial role in the development of the disease. IL-23 activates immune cells to produce IL-17, which, along with other cytokines, causes excessive proliferation and impaired differentiation of keratinocytes. Psoriasis provides a well-characterized model for studying ncRNA-mediated inflammation and keratinocyte dysfunction [85]. It is the best-characterized human model of epidermal inflammation and keratinocyte dysfunction mediated by ncRNAs, allowing for detailed mechanistic analysis.

### 6.1. MicroRNAs

NcRNAs play an important role in the development of psoriasis. In particular, the pathogenic significance of miRNAs has been well established. For example, miR-21 inhibits apoptosis and promotes inflammation. Mir-31-5p (miR-31) and miR-203, on the other hand, stimulate keratinocyte hyperproliferation by regulating the cell cycle [86]. It has been shown that miR-31, which is highly abundant in psoriatic keratinocytes [87], inhibits glucose metabolism [88], leading to increased survival under glucose starvation. Interestingly, the same study also demonstrated that HaCaT keratinocytes expressing miR-31 tend to secrete cytokines and metabolites that promote the differentiation of CD4+ cells into Th17 cells [88].

A recent study has shown that miR-31-3p also regulates the barrier function by interacting with CLDN8 (Claudin-8) in psoriasis [89]. Differential expression of miRNAs between normal and psoriatic keratinocytes may have potential for diagnostic use. For instance, miR-21-5p, together with miR155-5p and miR-16-5p, is highly expressed in extracellular vesicles from patients with psoriasis. This suggests that miRNA data could be used as diagnostic markers for this condition [90]. A recent study found that miR-202-5p, miR-146a, and miR-508 are highly expressed in psoriatic lesions, but not in healthy skin cells. Additionally, psoriatic keratinocytes have reduced expression of miR-200b, miR-141, and miR-429, which are all members of the miR–200 family. This family of miRNAs is highly expressed in skin and plays an important role in maintaining skin homeostasis [91,92]. Another study showed a decrease in the expression of miR-9, miR-133a-3p, and miR-375 in psoriasis keratinocytes [93]. Together, these findings highlight that both upregulated and downregulated miRNAs contribute to psoriatic pathology via dysregulation of keratinocyte fate decisions.

### 6.2. LncRNAs

LncRNAs involved in the pathogenesis of the disease include UCA1 (Urothelial cancer-associated 1), whose expression positively correlates with pro-inflammatory factors such as IL-6 and CXCL1 (C-X-C motif chemokine ligand 1) [94]. The recently discovered lncRNA CYDAER (Cytokine-induced differentiation-associated epidermal RNA) is overexpressed in psoriatic keratinocytes, which are induced by the cytokine IL-17A. Knockdown of CYDAER leads to the terminal differentiation of keratinocytes [85].

LncRNA MEG3 (Maternally expressed gene 3), which is known for its ability to inhibit cell proliferation, is significantly reduced in psoriatic lesions, making it a potential diagnostic marker for this condition [95]. Other well-known lncRNAs that are associated with psoriasis include MALAT-1, ANRIL, PRINS, XIST, and GAS5, among others [40,96].

Keratinocytes in psoriasis and other inflammatory skin conditions are characterized by the high expression of certain.

**Figure 3:**
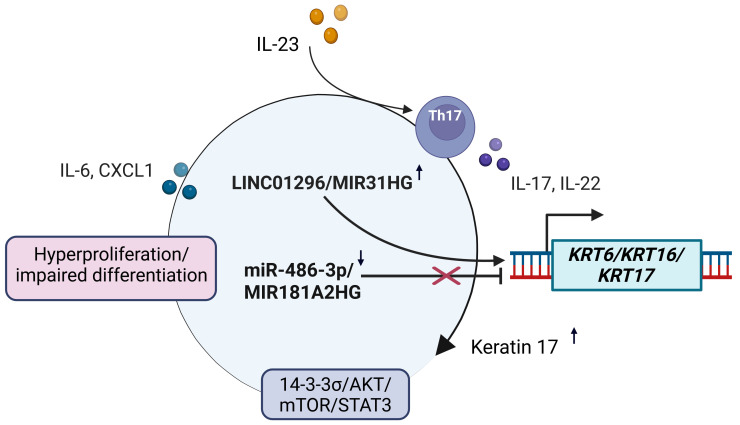
A crosstalk between non-coding RNAs (ncRNAs) associated with psoriasis and “stress-keratins” K6/K16/K17. Mir-486-3p, which directly targets *KRT17*, was found to be downregulated in psoriatic keratinocytes. This downregulation facilitates the expression of keratin 17 (K17) and the feedback loop between K17 and Th17 cells. Similarly, the expression of long non-coding RNA MIR181A2HG is downregulated in psoriatic keratinocytes and correlates negatively with K6/K16 expression. On the other hand, LINC01296 and MIR31HG are upregulated in psoriatic keratinocytes, and their expression correlates positively with K17 and K6/K16, respectively.

A crosstalk between non-coding RNAs (ncRNAs) associated with psoriasis and “stress keratins” K6, K16, and K17 (encoded by the genes *KRT6*, *KRT16* and *KRT17*, respectively) [4,59,97]. This group of intermediate filaments has a unique position among other cytokeratins, as unlike other IFs, their functions are not solely structural [4,59]. Keratins K6, K16, and K17 are not present in normal epidermal cells, but they are highly expressed in psoriatic lesions. These keratins play important roles in regulating cell migration, mitochondrial morphology, and redox balance within the cell. Keratins 6 and 16 help control these processes, while keratin 17 acts as a driver for cell growth and proliferation through the 14-3-3σ/AKT/mTOR/STAT3 signaling pathway [59,98,99]. The expression of these keratins is mainly regulated by pro-inflammatory cytokines, such as Th1, Th17, and Th22, through the activation of AP-1 and Nrf2. Keratin 17, in turn, stimulates the production of these cytokines. In psoriasis, this regulatory mechanism forms a feedback loop that stimulates keratinocyte growth and proliferation, disrupting the normal epidermal differentiation process [4,59,100]. Thus, keratins K6, K16 and K17 are important regulators of epidermal homeostasis. Therefore, their interaction with various non-coding RNAs is of interest. Importantly, several psoriasis-associated ncRNAs converge on K17 regulation, creating a focal point for therapeutic targeting (Figure 3). In a study by Jiang et al., the expression of miR-486-3p was shown to be markedly suppressed in psoriatic keratinocytes. Additionally, the level of miR-486-3p correlated negatively with the severity of the disease and the size of psoriatic lesions [101]. The authors also demonstrated that K17 is a direct target of miR-486-3p, while miR-486-3p itself is regulated by the TGFβ signaling pathway. Specifically, SMAD2/3 enhances the processing of miR-486-3p [101]. Interestingly, miRNA-485-5p has been found to be involved in the suppression of K17 in pancreatic and oral cancer cases [102,103]. It also reduces the proliferative and migratory activity of keratinocytes in the HaCaT cell line [104]. Another study sheds light on the role of LINC01296, which has been found to be overexpressed in biopsy samples taken from patients with psoriasis [105]. According to the results of the study, the knockdown of LINC01296 led to a significant reduction in the levels of *KRT6*, *KRT16*, and *KRT17* [105]. Another lncRNA, whose expression is reduced in psoriatic keratinocytes, is MIR181A2HG [106]. It was shown that suppression of this lncRNA leads to activation of K6 and K17 at the protein and mRNA levels, whereas overexpression of MIR181A2HG led to the opposite effect [106]. Gao and co-authors found that lncRNA MIR31HG is highly expressed in psoriatic keratinocytes and showed that knockdown of MIR31HG leads to a decrease in proliferation in keratinocytes of HaCaT. In addition, inactivation of MIR31HG led to a decrease in *KRT6* and *KRT16* levels [107]. Together, these findings indicate that lncRNAs and miRNAs cooperate to amplify the K17-centered proliferative and inflammatory circuit in psoriasis.

### 6.3. CircRNAs

The role of circular RNAs in the pathogenesis of psoriasis is still not well understood, despite the increasing number of studies indicating their involvement in this disease. One recent study, for example, found that the well-known circRNA CDR1 (cerebellar degeneration-related protein 1 antisense; also known as ciRS-7) is overexpressed in psoriatic skin and plays a role in regulating miR-7-5p and miR-135b-5p levels [108]. Another study found that circARNTL2 is also increased in psoriatic lesions, where it promotes keratinocyte proliferation by positively regulating Serpin B4 (Serpin family B member 4) [109]. Another example is circRNA Circ0061012, which is highly expressed in psoriasis but not in healthy skin. This circRNA promotes the progression of psoriasis by activating Ki67 and MMP9 through the miR-194-5p/GAB1 (GRB2-associated-binding protein 1) axis [105,110,111]. These findings further support the contribution of circRNA networks to keratinocyte hyperactivation. Seeler and colleagues demonstrated that treatment with secukinumab for psoriasis (which inhibits IL-17A) leads to significant changes in the profile of circulating RNAs. Additionally, they found that exposure to secukinumab results in a significant decrease in the expression of ciRS-7 and circPTPRA, which correlates directly with the severity of the condition [112].

Collectively, dysregulated circRNAs contribute to psoriatic inflammation and hyperproliferation, complementing miRNA- and lncRNA-driven signaling pathways highlighted in Figure 3.

Thus, an increasing number of studies highlight the significance of non-coding RNAs in the development of psoriasis. Meanwhile, understanding the role of specific ncRNAs could lead to new therapies for the disease.

## 7. NcRNAs as Potential Therapeutic Agents and Targets

**Figure 4:**
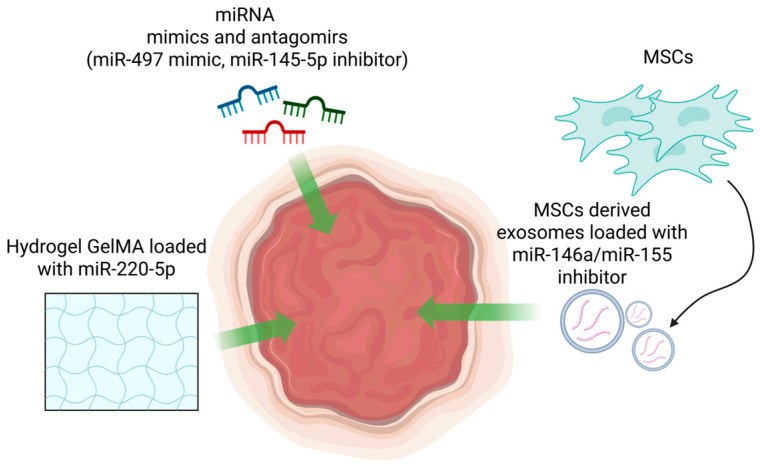
MicroRNA (miRNA)-based therapeutic approaches: injection of mimetics and inhibitors of miRNAs (e.g., miR-497 mimic, miR-145-5p inhibitor), exosomes derived from mesenchymal stem cells (MSCs) loaded with miRNA mimics/inhibitors, and hydrogels such as GelMA with miR-223-5p for improved healing processes.

The groundbreaking work by Fire and his colleagues that illuminated the mechanism of RNA interference became the starting point for exploring regulatory non-coding RNAs [113]. As our understanding of different types of ncRNA in cells has increased, so has the idea that they can be targeted for treating various diseases (Figure 4). Non-coding RNAs themselves can also be used as therapeutic agents. For instance, antagomirs or anti-microRNAs (ASOs) are small chemically synthesized RNAs that covalently bind to and inhibit the activity of specific miRNAs. These can be used in various tissues without causing an immune response. The use of ASO targeting lncRNAs and the inhibition of pathogenic circRNAs provides a basis for targeted therapy for a number of diseases [114,115,116]. In particular, in psoriasis, pro-inflammatory and proliferative roles of miR-17, miR-383 and miR-155 microRNAs have been identified. The modulation of these miRNAs can weaken hyperproliferation and inflammation, making them promising targets for anti-miRNA therapy [117,118,119]. Transcripts involved in the regulation of keratinocyte cell cycle, such as LINC01026 and MIR181A2HG, have been identified in patients with psoriasis. The use of ASOs, particularly gapmers, has shown potential for inhibiting these transcripts [105,106,120]. Molecules that enhance keratinocyte proliferation, such as circARNTL2, have been identified among the circRNA class. Their knockout is also being considered as a potential therapeutic strategy [109]. In addition, lncRNAs and miRNAs are found in extracellular vesicles released by keratinocytes and play a role in immune responses. For example, the lncRNA PRKCQ-AS1 has been shown to enhance Th17 cell differentiation [121]. Therapeutic approaches can be applied not only within target cells, but also at the level of their interaction with other cell types. In particular, the use of “sponges” for miRNAs, which are complementary oligonucleotides, allows for intercepting free ncRNAs before they enter the cell. Additionally, the use of therapeutic ncRNAs, such as anti-miRNAs and mimics, can help regulate the balance between immune cells and keratinocytes. Preclinical models of psoriasis have shown promising results, with targeted suppression of key genes involved in the disease using siRNAs leading to improved signs of the condition (for example, suppression of Fn14 siRNAs improved PASI-like indicators in animal models) [122,123].

NcRNAs that directly regulate the functions of keratinocytes and are responsible for the integrity and functionality of the skin barrier have been described in atopic dermatitis. Specifically, miR-10a-5p has been shown to inhibit keratinocyte proliferation and is linked to impaired barrier function. MiR-146a and miR-939, on the other hand, modify and enhance innate inflammatory responses [124,125,126]. As a result, these non-coding RNAs are seen as potential targets for localized therapy using microRNAs.

Wound healing is a separate area of research in ncRNA-based therapy. CircRNAs, in particular, are able to “protect” functional miRNAs in the skin and accelerate repair, potentially making them useful in the treatment of skin conditions. Therapeutic strategies for treating diabetic wounds include the use of targeted oligonucleotides and miRNA mimics, as well as smart carriers. Injections of miR-497 mimics have been shown to improve wound closure in diabetic mice. Inhibition of miR-145-5p has been shown to relieve PDGF-D blockage and accelerate repair—two clinically promising approaches (Figure 4) [76].

MSC-derived exosomes have been shown to reduce oxidative stress and normalize mitochondrial function in high glucose environments. They also promote healing in mouse models of diabetes, making them a promising therapeutic option [127]. MSCs-derived exosomes can also be loaded with miRNAs or ASOs. Particularly, Li and co-authors report that engineered exosomes loaded with miR-146a have improved therapeutic potential for diabetic wound healing [128]. In line with this data, a similar effect has been shown for MSCs-derived exosomes loaded with an anti-miR-155 ASO [129].

At the same time, the delivery of ncRNAs in smart hydrogels is being developed. For example, a GelMA hydrogel that carries the miR-223-5p (miR-223*) mimic has been shown to convert macrophages into the M2 phenotype, speeding up the reduction in inflammation and epithelialization in a mouse model [130]. In general, the hydrogel protects the unstable oligonucleotide and provides local, prolonged exposure.

In epithelial skin tumors, particularly squamous cell carcinomas (SCCs), ncRNAs are mostly used as biomarkers in clinical practice. However, functional studies have confirmed the role of certain miRNAs, such as miR-21, in the invasion and metastasis of keratinocyte tumors. These studies have also outlined directions for anti-miRNA therapies [131,132,133]. Recently, there has been an increasing amount of data confirming the potential of miRNAs and lncRNAs as diagnostic and prognostic biomarkers and future therapeutic targets (Table 1) [134].

Targeted delivery to the epidermis is crucial for clinical success. Non-invasive and minimally invasive methods are of particular interest, including topical compositions for transdermal siRNA delivery, ionic liquids, various nanoparticles, and microneedle patches, which improve penetration through the stratum corneum and provide targeted delivery to keratinocytes. Additionally, exosomes are being explored as potential carriers for skin-targeted ncRNA preparations [122,135,136].

However, successful clinical implementation requires addressing the following challenges: coordinating and confirming priority targets; ensuring safety in terms of the skin’s immune response; and bringing delivery systems to a stable and predictable level for real-world patients [137].

**Table 1:** Non-coding RNAs in epidermal differentiation: markers, targets, and therapeutic perspectives.

NcRNA	Subject/Model	Biological Effect	Confirmed Targets/Mechanism	Therapeutic Perspective	References
miR-17-3p	Psoriatic epidermis; human keratinocytes	Pro-proliferative and pro-inflammatory effects; shifts the program away from differentiation (↓ *IVL*/*FLG* as a consequence of the proliferative shift)	Targets CTR9; increases cell proliferation and cytokine secretion	Use anti-miR-17-3p ASO to inhibit the proliferation of keratinocytes	[117]
miR-383	Psoriasis model; keratinocytes	The anti-inflammatory effect and stimulation of cell differentiation through suppression of JAK/STAT activity	Directly targeting LCN2 → JAK3/STAT3 signals	Simulating the action of miR-383 to help restore balance in the differentiation process	[118]
miR-155	Human keratinocytes; psoriatic skin	A reduction in miR-155 levels is necessary for normal cell differentiation and barrier formation (↑ *IVL*, *FLG*, *KRT1*, and *KRT10*)	It is high in psoriatic plaque; the decrease supports the differentiation of CC	Anti-miR-155 is designed to help normalize differentiation processes and create a barrier	[119]
LINC01026	Psoriatic keratinocytes	Pro-proliferative effect; inhibits the transition to differentiation	Increases EHF and promotes cell cycle progression	Anti-LINC01026 ASO to reduce hyperproliferation	[105]
MIR181A2HG	Psoriatic keratinocytes, HaCaT keratinocytes	Anti-proliferative; promotes the differentiation pathway	The miR-223-3p/SOX6 axis and SRSF1 binding are associated with suppression of proliferation	lncRNA replacement therapy or anti-miR-223-3p to restore the miR181A2HG axis	[8,9]
circARNTL2	Psoriatic keratinocytes	Pro-proliferative; shifts the balance toward proliferation	Stimulates the progression of the keratinocyte cell cycle	ASO/Cas13 against circARNTL2 for promoting differentiation	[109]
PRKCQ-AS1	Keratinocyte exosomes /CD4+ T cells (psoriasis)	Increases inflammation caused by Th17 cells, which in turn negatively affects the differentiation process	Activates STAT3 and promotes Th17 differentiation in recipient cells	Application of ASO to PRKCQ-AS1 (PRKCQ-AS1 release and capture block)	[121]
Anti-Fn14 siRNA (targets TNFRSF12A)	Keratinocytes; psoriatic mouse skin	↓ hyperproliferation; improvement of PASI-like metrics → normalization of differentiation	Fn14 siRNA knockdown; transdermal ionic liquid delivery	Topical/Transdermal siRNA therapy for Fn14 nodes	[122,138]
miR-146a	Psoriatic keratinocytes	It inhibits the activity of the natural NF-kB response, indirectly promoting differentiation	IRAK1/TRAF6 weakens IL-17/TLR-dependent responses	Mimic miR-146a to reduce inflammation and restore epidermal barrier	[126]
miR-21	Psoriatic keratinocytes, aging/wounded skin	Increased miR-21 inhibits CC apoptosis and differentiation, and is associated with the caspase-8 pathway. It also affects SATB1	CASP8 (psoriasis) and SATB1 (skin aging)	Anti-miR-21 is used to restore the balance between apoptosis and differentiation	[131,132,133]
miR-10a-5p	Atopic dermatitis, keratinocytes	Modulates cell proliferation and differentiation; blood pressure is elevated in certain areas	Regulated by the inflammatory environment and affects barrier and cell cycle genes	Anti-miR-10a-5p is used to normalize differentiation in blood pressure	[125]
miR-939	Atopic dermatitis, keratinocytes	Pro-inflammatory cytokines in the CC (MMP1/3/9 and ICAM1) indirectly worsen the barrier and differentiation	Increases the activity of matrix metalloproteinases (MMPs), leading to increased inflammatory processes	Anti-miR-939 is used to reduce inflammation and protect the skin barrier	[139]

## 8. Challenges, Limitations, and Future Directions

Since ncRNAs have been proposed as potential therapeutic agents and targets, research in this field has been ongoing for decades. As a result, several ncRNA-based therapies for various diseases of the eye, liver, muscle, and central nervous system have been approved by the FDA and the European Medicines Agency [140]. Skin, being the most accessible organ, continues to be a preferred target for drug discovery. This includes ncRNA-based therapies for a variety of skin conditions, ranging from inflammatory diseases to hypertrophic scars and keloids [141]. Despite established clinical potential, there remain significant challenges and limitations in translating ncRNA-based therapeutics into dermatology:

- Target screening and identification are crucial for the development of ncRNA-based therapeutics. Despite the discovery of dozens of potential therapeutic agents in the last decade, only a limited number of ncRNA treatments have progressed to clinical trials. Recently, screening strategies have incorporated cutting-edge technologies such as high-throughput screening (HTS), small molecule microarrays (SMMs), and fragment-based drug discovery (FBDD) [142];
- Efficient transdermal drug delivery remains a challenge due to the barrier function of the skin and intracellular RNA degradation. Various strategies have been developed, including microneedles, electroporation, nanoparticles, hydrogel systems, and liposomes. However, each of these methods requires further optimization and validation for new drug candidates [143,144];
- Since ncRNAs can regulate multiple pathways simultaneously, off-target effects remain a significant concern for clinical applications. Therapeutic oligonucleotides can impact pathways unrelated to the intended target and influence immune responses. Chemical modifications and other strategies can help to reduce these effects, but careful safety evaluation is still essential [144].

Currently, ncRNAs have great potential as diagnostic tools, with a very limited number of ncRNA-based therapies having reached clinical trial stages. However, the field of applied ncRNA therapeutics is flourishing, providing novel drug candidates, new approaches to overcome limitations and challenges, and insights into fundamental RNA biology.

## Data Availability

No new data were created or analyzed in this study. Data sharing is not applicable to this article.
